# Linking Ventilation Heterogeneity Quantified via Hyperpolarized ^3^He MRI to Dynamic Lung Mechanics and Airway Hyperresponsiveness

**DOI:** 10.1371/journal.pone.0142738

**Published:** 2015-11-16

**Authors:** Justin K. Lui, Harikrishnan Parameswaran, Mitchell S. Albert, Kenneth R. Lutchen

**Affiliations:** 1 Boston University, School of Medicine, Boston, MA, United States of America; 2 Department of Biomedical Engineering, Boston University, Boston, MA, United States of America; 3 Department of Radiology, Brigham and Women’s Hospital, Boston, MA, United States of America; 4 Department of Chemistry, Lakehead University, Thunder Bay, ON, Canada; 5 Thunder Bay Regional Research Institute, Thunder Bay, ON, Canada; Technion - Israel Institute of Technology, ISRAEL

## Abstract

Advancements in hyperpolarized helium-3 MRI (HP ^3^He-MRI) have introduced the ability to render and quantify ventilation patterns throughout the anatomic regions of the lung. The goal of this study was to establish how ventilation heterogeneity relates to the dynamic changes in mechanical lung function and airway hyperresponsiveness in asthmatic subjects. In four healthy and nine mild-to-moderate asthmatic subjects, we measured dynamic lung resistance and lung elastance from 0.1 to 8 Hz via a broadband ventilation waveform technique. We quantified ventilation heterogeneity using a recently developed coefficient of variation method from HP ^3^He-MRI imaging. Dynamic lung mechanics and imaging were performed at baseline, post-challenge, and after a series of five deep inspirations. AHR was measured via the concentration of agonist that elicits a 20% decrease in the subject’s forced expiratory volume in one second compared to baseline (PC_20_) dose. The ventilation coefficient of variation was correlated to low-frequency lung resistance (*R* = 0.647, *P* < 0.0001), the difference between high and low frequency lung resistance (*R* = 0.668, *P* < 0.0001), and low-frequency lung elastance (*R* = 0.547, *P* = 0.0003). In asthmatic subjects with PC_20_ values <25 mg/mL, the coefficient of variation at baseline exhibited a strong negative trend (*R* = -0.798, *P* = 0.02) to PC_20_ dose. Our findings were consistent with the notion of peripheral rather than central involvement of ventilation heterogeneity. Also, the degree of AHR appears to be dependent on the degree to which baseline airway constriction creates baseline ventilation heterogeneity. HP ^3^He-MRI imaging may be a powerful predictor of the degree of AHR and in tracking the efficacy of therapy.

## Introduction

Asthma is a disease characterized by airway hyperresponsiveness (AHR) leading to bronchoconstriction and airway inflammation, which result in ventilation deficiencies throughout the lungs. Although still incompletely understood, AHR tends to occur more in people that have a diminished capacity to maximally dilate their airways with a deep inspiration (DI) [1–6]. Also, prohibiting DIs for extended periods of time seems to enhance AHR [7, 8] and increase airway narrowing in healthy individuals [9, 10]. Additionally, when exposed to bronchial provocation, airways bronchodilate following DIs [5] in healthy subjects with the degree of recovery decreasing with asthma severity [11].

Recent studies have found that AHR is also related to the degree of ventilation heterogeneity at baseline [12, 13]. However, these studies employed an indirect method of quantifying ventilation heterogeneity using a multi-breath nitrogen washout (MNBW) technique [12–16]. The MBNW technique provides neither spatial nor specific regional information. Furthermore, recent modeling studies have questioned the ability of this technique to detect poorly ventilated or completely non-ventilated areas [17]. On the other hand, traditional methods of direct visualization such as computed tomography (CT) imaging [18] and positron emission tomography (PET) [19–22] require invasive radiation exposure making them unsuitable for clinical evaluation of ventilation heterogeneity. Recently, Hyperpolarized Helium-3 MRI (HP ^3^He-MRI) has emerged as a novel imaging modality for probing airway conditions in a number of obstructive pulmonary diseases [23–32]. In addition to its lack of radiation exposure, it provides a direct visualization of ventilation enabling quantification of lung volumes [28, 30] as well as characterization of ventilation distributions [23, 32] and applications to computational modeling [23].

Indirect methods of quantifying baseline ventilation heterogeneity via analysis of MBNW have shown a relationship to AHR [12, 13]. Direct lung imaging, in principal, can be used to spatially identify and quantify ventilation heterogeneity. The goal of this study was to use our advances in imaging analysis to establish a quantitative relationship between ventilation heterogeneity and the dynamic changes in lung mechanical function and airway hyperresponsiveness in asthmatic subjects. To this end, here we introduce and apply the coefficient of variation (CV) of ventilated volume fraction (VVF), evaluated from static HP ^3^He-MRI images, as a measure of ventilation heterogeneity, and we correlate this index with measurements of dynamic lung mechanics and AHR in healthy and asthmatic subjects.

## Materials and Methods

### Subject Enrollment and Experimental Protocol

The research protocol used in this study was approved by both Boston University and Brigham and Women’s Hospital Institutional Review Boards. Written informed consent was obtained from all recruits, which consisted of four healthy subjects and nine asthmatic subjects. Before the day of the study visit, airway reactivity was assessed in all subjects by interpolating a methacholine (MCh) dose-response curve to obtain the concentration of agonist that elicits a 20% decrease in the subject’s forced expiratory volume in one second (FEV_1_) compared to baseline (PC_20_). Doses used to determine the PC_20_ did not exceed a maximum dose of 25.0 mg/ml. For our protocol, healthy subjects were nonsmokers with no history of respiratory diseases and exhibited PC_20_ values of >25 mg/mL. Mild-to-moderate asthmatics were defined as physician-diagnosed asthmatics exhibiting a FEV_1_ ≥ 60% predicted, without inhaled or oral corticosteroids, asthma symptoms less than 7 times per week requiring β_2_-agonist use, and well-controlled on low dose regimen of inhaled corticosteroids. The demographics are detailed in Table 1.

**Table 1:** Subject demographics.

	Subject	Gender	Age, yr	Height, cm	Weight, kg	FEV_1_, %pred	PC_20_ MCh (mg/mL)
**Healthy**							
	H1	M	22	180	91	100	>25
	H2	F	25	150	53	108	>25
	H3	F	24	165	54	115	>25
	H4	M	47	180	100	117	>25
**Asthmatic**							
	A1	F	32	173	70	90	10.60
	A2	F	38	160	69	76	>25
	A3	M	31	165	66	83	4.10
	A4	F	30	160	48	64	0.80
	A5	M	36	183	100	85	7.99
	A6	F	45	180	86	85	0.78
	A7	M	21	178	81	84	0.27
	A8	F	29	168	58	84	1.25
	A9	M	30	173	82	68	12.47

**Fig 1:**
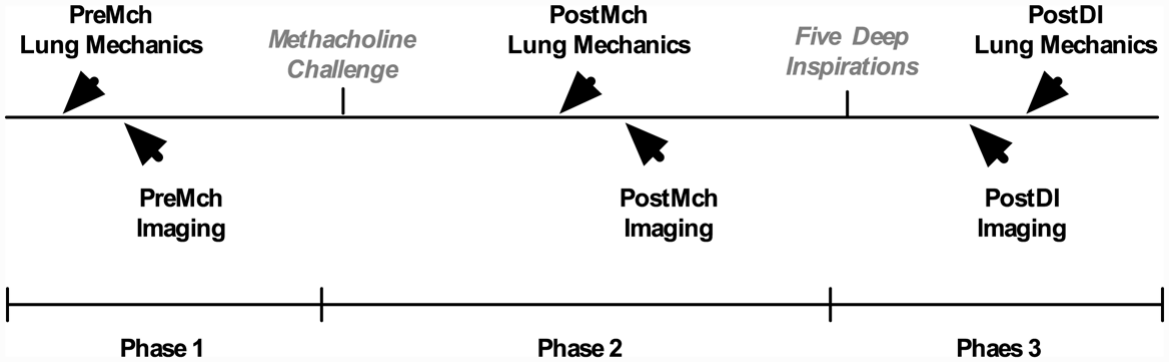
Experimental protocol. The experiments span three phases: phase I comprises lung mechanics and imaging, PreMch, followed by phase II which entails a Mch-challenge and lung mechanics and imaging, PostMch, and finally phase III which entails lung mechanics measurements and imaging, PostDI.

An on-site Pulmonologist monitored all our measurements. First, baseline spirometry (Micro Medical: Microlab 3000 series or Microloop 3535S) was measured from all subjects, followed by placement of an esophageal balloon catheter to estimate intrapleural pressure. Baseline (PreMch) lung mechanics were then measured following which baseline HP ^3^He-MRI images were acquired. Thereafter, a MCh-challenge (S&M Instrument Company, Inc., DSM2030 Dosimeter and DeVilbiss 646 Nebulizer) was administered to induce bronchoconstriction. The MCh concentrations were diluent (0.078 mg/ml, 0.156 mg/ml, 0.3125 mg/ml, 0.625 mg/ml, 1.25 mg/ml, 2.5 mg/ml, 5.0 mg/ml, 10.0 mg/ml, and 25.0 mg/ml). Every other MCh concentration in the challenge could be skipped until 10.0 mg/ml for healthy subjects or until the concentration one order of magnitude below the asthmatic subject’s known PC_20_ dose measured on screening day. This was due to time constraints with the polarization of HP ^3^He. The MCh challenge ended upon eliciting an FEV_1_ decrease of 20% or the 25.0 mg/ml dose. Following the MCh-challenge (PostMch), subjects were asked to refrain from taking any deep inspirations (DIs) until otherwise instructed. Lung mechanics were measured again, and HP ^3^He-MRI images were reacquired. Finally, the subject was instructed to sit in an upright position and take five DIs (PostDI) after which they were reinserted into the MRI for the final set of image acquisition. Lung mechanics measurements were then subsequently repeated. A schematic of the protocol is illustrated in Fig 1.

### Dynamic Lung Mechanics Measurements

Dynamic lung resistance (*R*_*L*_) and lung elastance (*E*_*L*_) were measured using the Optimal Ventilation Waveform (OVW) technique, as previously described [33–35]. Briefly, the technique entails delivering a waveform via a linear motor at seven non-sum, non-difference frequencies ranging from 0.1 to 8 Hz and with phases optimized such that the forcing signal also ventilates the subject with a typical tidal volume per breath. In a seated position, each subject was instructed to relax to allow the ventilator to deliver the OVW over a period of 40–60 seconds. Flow (*Q*_*ao*_) and pressure (*P*_*ao*_) were measured at the airway opening, and an esophageal pressure (*P*_*es*_) was measured via a balloon catheter from which a transpulmonary pressure (*P*_*tp*_) can be estimated from the difference between *P*_*ao*_ and *P*_*es*_. A lung impedance (*Z*_*L*_ *(ω)*) (Eq 1) can be calculated in which *R*_*L*_ would result from the real or in-phase component (Eq 2) and *E*_*L*_ would result from the imaginary or out-of-phase component (Eq 3) in the frequency domain.

$$Z_{L}(\omega )=P_{tp}(\omega )/Q_{ao}(\omega )$$ (1)

$$R_{L}=\text{Re}[Z_{L}(\omega )]$$ (2)

$$E_{L}=-\omega (\text{Im}[Z_{L}(\omega )])$$ (3)

From the OVW data we extracted several features that have been shown to be sensitive to airway and tissue conditions and the pattern of airway constriction. Low frequency (0.1 Hz) lung resistance (R_low_) represents the combined impact of viscoelastic tissues and any heterogeneous constriction pattern that might emerge post-challenge. At low frequencies, E_L_, approximates the “quasi-static” tissue elastance of the amount of lung that is ventilated at that frequency neglecting any inertive components due to the accelerative motion of the air in the lung which becomes important at higher frequencies. Thus, low frequency lung elastance, E_low_, can increase either due to an inherent increase in tissue stiffness or because of heterogeneous airway constriction preventing substantive portion of the lung tissue from being ventilated (e.g., a smaller lung). High frequency (8 Hz) lung resistance (R_high_) reflects primarily the net overall conductive airway resistance of the airway tree alone [33, 34]. The frequency dependence (R_het_) is calculated from the difference between R_low_ and R_high_ and reflects the impact of heterogeneous constriction [3, 36].

### Image Acquisition

For our image acquisition, each subject was instructed to inhale a ~1 liter mixture of 33% HP ^3^He and 67% N_2_ from functional residual capacity (FRC). Subjects were instructed to try to inhale the 1 L of gas within a 5- to 8-second period with each scan spanning a total of about 11 seconds. Images were acquired on a General Electric Signa LX 1.5 MRI scanner equipped with a heterodyne system which included frequency mixers to image at the ^3^He NMR frequency of 48.65 Hz. The system interfaced with a flexible quadrature lung coil (Clinical MR Solutions, Brookfield, WI) tuned to the same frequency. Hyperpolarization of the ^3^He gas was initiated through a collision spin exchange with vaporized Rubidium optically pumped using a custom-built polarizer. The scans employed a Fast Gradient Echo pulse sequence that compiled coronal multi-slice images with a field of view of 46 cm, 128 x 256 matrix dimensions (zero-padded to 256 x 256), 13-mm slice thickness, 0-mm gap between slices, 1.8-mm in-slice resolution, 31.25-kHz bandwidth, 14–18°flip angle, TE/TR 1.228 ms/50-75 ms, and interleaved data acquisition. Subjects were instructed to hold still during image acquisition as small movements can affect the quality of images obtained. Approximately 8–14 slices were compiled for each subject depending on the anterior to posterior depth of the lung to capture the entire coronal lung field.

### Image Processing

Ventilated airspaces were segmented through a recently developed semiautomatic algorithm described elsewhere [30]. Briefly, it comprises a three step process involving a statistical denoising scheme, followed by a clustering of ventilation classes, and finally a removal of the trachea and the major airways. The remaining non-background pixel intensities were then correspondingly adjusted for signal bias as:

$${\hat{S}}_{i,j,k}=\sqrt{S_{i,j,k}^{2}-\frac{2}{\pi }{\bar{S}}_{BG}^{2}}$$ (4)

where ${\hat{S}}_{i,j,k}$ is the corrected pixel intensity, ${\bar{S}}_{BG}$ is the average pixel intensity of the sampled noise region, *i* and *j* are the pixel indices and *k* is the slice index [37, 38]. The sum of the signal intensities, $\sum_{i,j,k}{\hat{S}}_{i,j,k}$, represent the amount of inhaled gas. By calculating a fraction of each corresponding voxel, ${\hat{S}}_{i,j,k}$, we obtain a fraction of inhaled gas, termed ventilated volume fraction (*VVF*_*i*,*j*,*k*_)[23, 32] given as:

$$VVF_{i,j,k}=\frac{{\hat{S}}_{i,j,k}}{\sum_{i,j,k}{\hat{S}}_{i,j,k}}$$ (5)

A corresponding CV of the VVF was calculated from the standard deviation of VVF (σ_*VVF*_) and the mean VVF (*μ*_*VVF*_) for each condition, PreMch, PostMch, and PostDI as follows:

$$CV_{VVF}=\frac{\sigma_{VVF}}{\mu_{VVF}}$$ (6)

### Statistical Analysis

Data was collected and HP ^3^He-MRI images were analyzed through a self-developed image processing algorithm on MATLAB (MathWorks Inc., MA). Linear regressions and statistical analyses were obtained through SigmaPlot (Systat Software, Inc., CA). A paired two-tailed student’s *t*-test was used to assess for significant differences between each condition (PreMch, PostMch, PostDI), and an unpaired two-tailed student’s *t*-test was used to assess for significant differences between each subgroup (healthy and asthmatics). A probability of *P* < 0.05 with a statistical power ≥ 0.8 was considered statistically significant.

## Results

**Fig 2:**
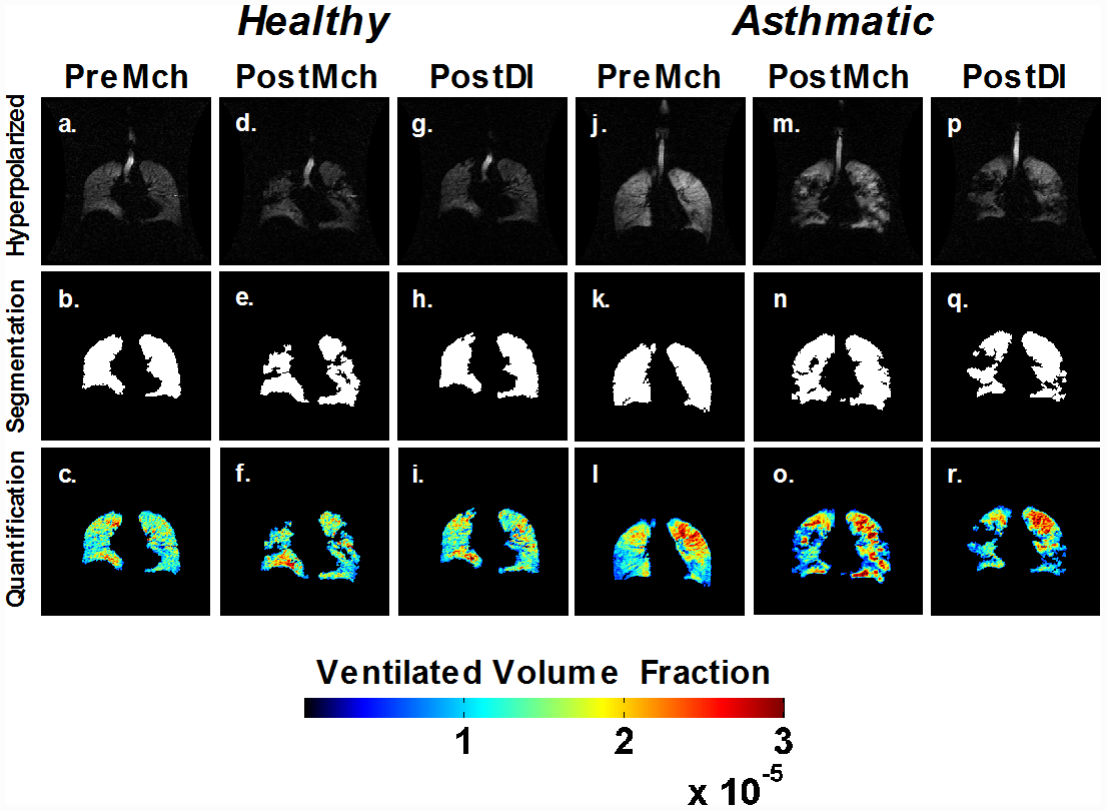
Single slice ventilation images for a healthy and asthmatic subject for PreMch, PostMch, and PostDI. HP ^3^He-MRI images (top row) are first segmented to evaluate for ventilated regions (middle row) and quantified to determine a mapped-out VVF (bottom row). Note the increased pockets of elevated ventilation in both healthy and asthmatics following a Mch-challenge and the lack of recovery for the asthmatic after a series of DIs.

**Fig 3:**
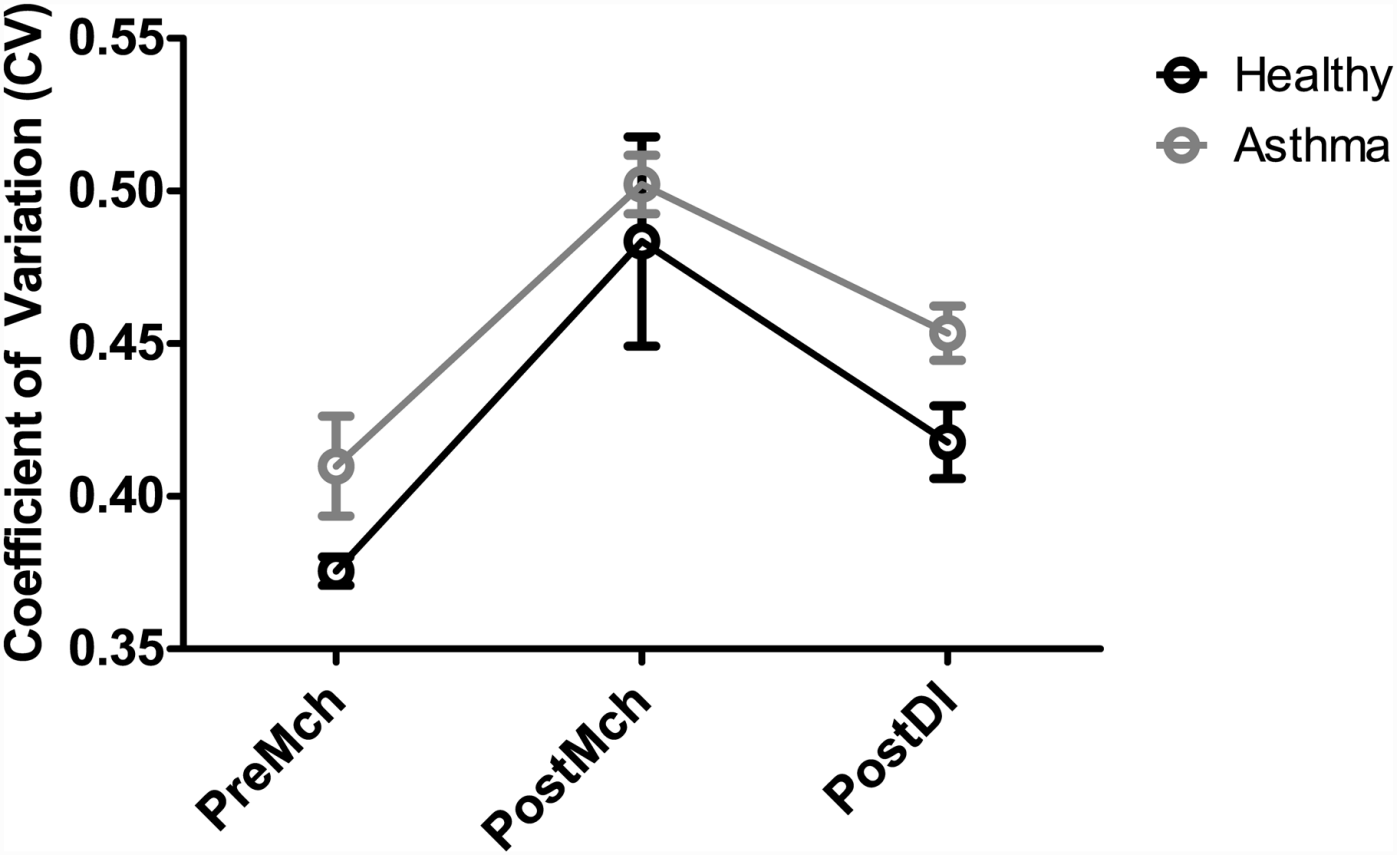
CV for PreMch, PostMch, and PostDI conditions with corresponding standard errors. Note across all three conditions, the mean CVs for the asthmatic subjects were consistently higher than the mean CVs for the healthy subjects, although not statistically significant. Refer to Tables 2 and 3 for statistical significance in comparisons between PreMch, PostMch, and PostDI.

Patchy areas of high and very low ventilation are evident following induced bronchoconstriction in both healthy and asthmatic subjects (Fig 2). Following a series of DIs whereas ventilation recovery visually occurs in the healthy subject, areas of poor ventilation are still prevalent in the asthmatic subject. A corresponding CV was calculated and a summary is shown in Fig 3 and in Tables 2 and 3. PreMch healthy subjects maintained a CV of 0.38 ± 0.01 (mean ± SD) which increased to 0.48 ± 0.07, PostMch, and recovered to 0.42 ± 0.02, PostDI. PreMch asthmatic subjects maintained a CV of 0.42 ± 0.05 which increased to 0.51 ± 0.03, PostMch, and recovered only partially to 0.46 ± 0.03, PostDI. However, there was not a statistically significant difference in the CVs PreMch, CVs PostMch, and CVs PostDI between the healthy and asthmatic subjects. In the asthmatic subjects, there was a statistically significant difference between CV PreMch and CV PostMch (*P* < 0.001), but not between CV PostMch and CV PostDI and between CV PreMch and CV PostDI. However, in the healthy subjects, there was not a statistically significant difference between CV PreMch and CV PostMch, between CV PostMch and CV PostDI, and between CV PreMch and PostDI. The results likely reflect the small number of healthy subjects.

**Table 2:** Dynamic lung mechanics and CV for healthy and asthmatic subjects.

	Condition	R_low_ (cm H_2_O/L/s) (mean ± SD)	R_high_ (cm H_2_O/L/s) (mean ± SD)	R_het_ (cm H_2_O/L/s) (mean ± SD)	E_low_ (cm H_2_O/L) (mean ± SD)	CV (mean ± SD)
**Healthy**						
	PreMch	3.72 ± 1.58	3.23 ± 1.10	0.49 ± 0.67	7.56 ± 1.93	0.38 ± 0.01^α^ ^,^ ^γ^
	PostMch	13.42 ± 8.90	5.25 ± 1.95	8.15 ± 6.98	14.19 ± 6.11	0.48 ± 0.07^α^
	PostDI	5.91 ± 2.70	4.55 ± 1.60	1.36 ± 1.41	6.31 ± 2.21	0.42 ± 0.02^γ^
**Asthmatic**						
	PreMch	6.75 ± 3.32^x^ ^,^ ^z^	5.10 ± 2.03^x^ ^,^ ^z^	1.66 ± 1.83^x^ ^,^ ^z^	9.06 ± 2.99^x^	0.42 ± 0.05^x^ ^,^ ^z^
	PostMch	18.35 ± 4.79^x^ ^,^ ^y^	8.31 ± 3.78^x^	10.04 ± 4.29^x^ ^,^ ^y^	17.49 ± 5.54^x^ ^,^ ^y^	0.51 ± 0.03^x^ ^,^ ^y^
	PostDI	12.87 ± 5.34^y^ ^,^ ^z^	7.77 ± 2.47^z^	5.09 ± 3.50^y^ ^,^ ^z^	12.08 ± 5.16^y^	0.46 ± 0.03^y^ ^,^ ^z^

^α^denotes statistically significant difference between PreMch and PostMch (*P* < 0.05) in healthy subjects.

^β^denotes statistically significant difference between PostMch and PostDI (*P* < 0.05) in healthy subjects.

^γ^denotes statistically significant difference between PreMch and PostDI (*P* < 0.05) in healthy subjects.

^x^denotes statistically significant difference between PreMch and PostMch (*P* < 0.05) in asthmatic subjects.

^y^denotes statistically significant difference between PostMch and PostDI (*P* < 0.05) in asthmatic subjects.

^z^denotes statistically significant difference between PreMch and PostDI (*P* < 0.05) in asthmatic subjects.

**Table 3:** Statistical Power for Pairwise Student’s *t*-tests.

	Condition	R_low_ (cm H_2_O/L/s) (mean ± SD)	R_high_ (cm H_2_O/L/s) (mean ± SD)	R_het_ (cm H_2_O/L/s) (mean ± SD)	E_low_ (cm H_2_O/L) (mean ± SD)	CV (mean ± SD)
Healthy						
	PreMch-PostMch	0.351	0.240	0.366	0.316	0.700*
	PostMch-PostDI	0.181	0.050	0.270	0.454	0.240
	PreMch-PostDI	0.127	0.578	0.069	0.398	0.756*
Asthmatic						
	PreMch-PostMch	1.000*	0.464*	1.000*	0.966*	0.997*
	PostMch-PostDI	0.483*	0.050	0.649*	0.418*	0.934*
	PreMch-PostDI	0.738*	0.578*	0.620*	0.178*	0.504*

*Corresponds to statistical power for where *P* < 0.05

**Fig 4:**
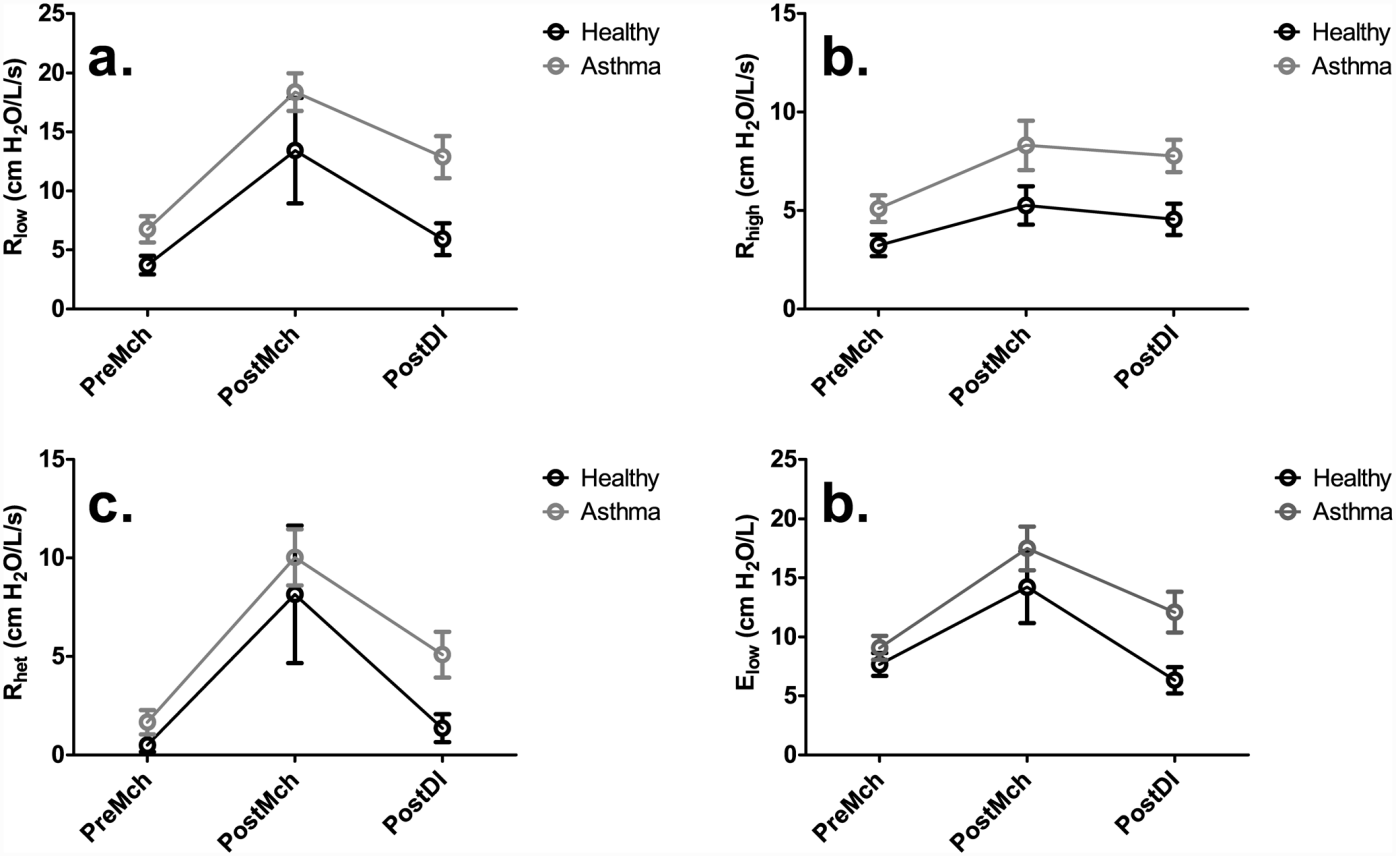
Dynamic lung mechanics measurements for PreMch, PostMch, and PostDI conditions, for R_low_ (a.), R_high_ (b.), R_het_ (c.), and E_low_ (d.) with corresponding standard errors. Again, across all three conditions, the mean values of all four indices of dynamic lung mechanics, R_low_, R_high_, R_het_, and E_low_ for the asthmatic subjects were consistently higher than those for the healthy subjects, although not statistically significant. Refer to Tables 2 and 3 for statistical significance in comparisons between PreMch, PostMch, and PostDI.

Dynamic lung mechanics for healthy and asthmatic subjects are summarized in Fig 4 and Tables 2 and 3. Under all three conditions, PreMch, PostMch, and PostDI, the mean values of all four parameters, R_low_, R_high_, R_het_, and E_low_, were higher in the asthmatic subjects in comparison to the healthy subjects, although not statistically significant. In the asthmatic subjects, there was a significant difference between PreMch and PostMch for indices of R_low_ (*P* < 0.001), R_het_ (*P* < 0.001), and E_low_ (*P* = 0.001), but not R_high_. There was not a significant difference in all four mechanical parameters (R_low_, R_high_, R_het_, and E_low_) between PostMch and PostDI and between PreMch and PostDI in both healthy and asthmatic subjects. Moreover, in the healthy subjects, there was also not a statistically significant difference between PreMch and PostMch. Interestingly, PostDI E_low_, an indicator of the heterogeneity of airway closures, returned to its mean baseline value in the healthy subjects but did not in the asthmatic subjects. However, the difference between E_low_ PostMch and E_low_ PostDI was not statistically significant in both healthy and asthmatic subjects.

### Relationship to Dynamic Lung Mechanics

**Fig 5:**
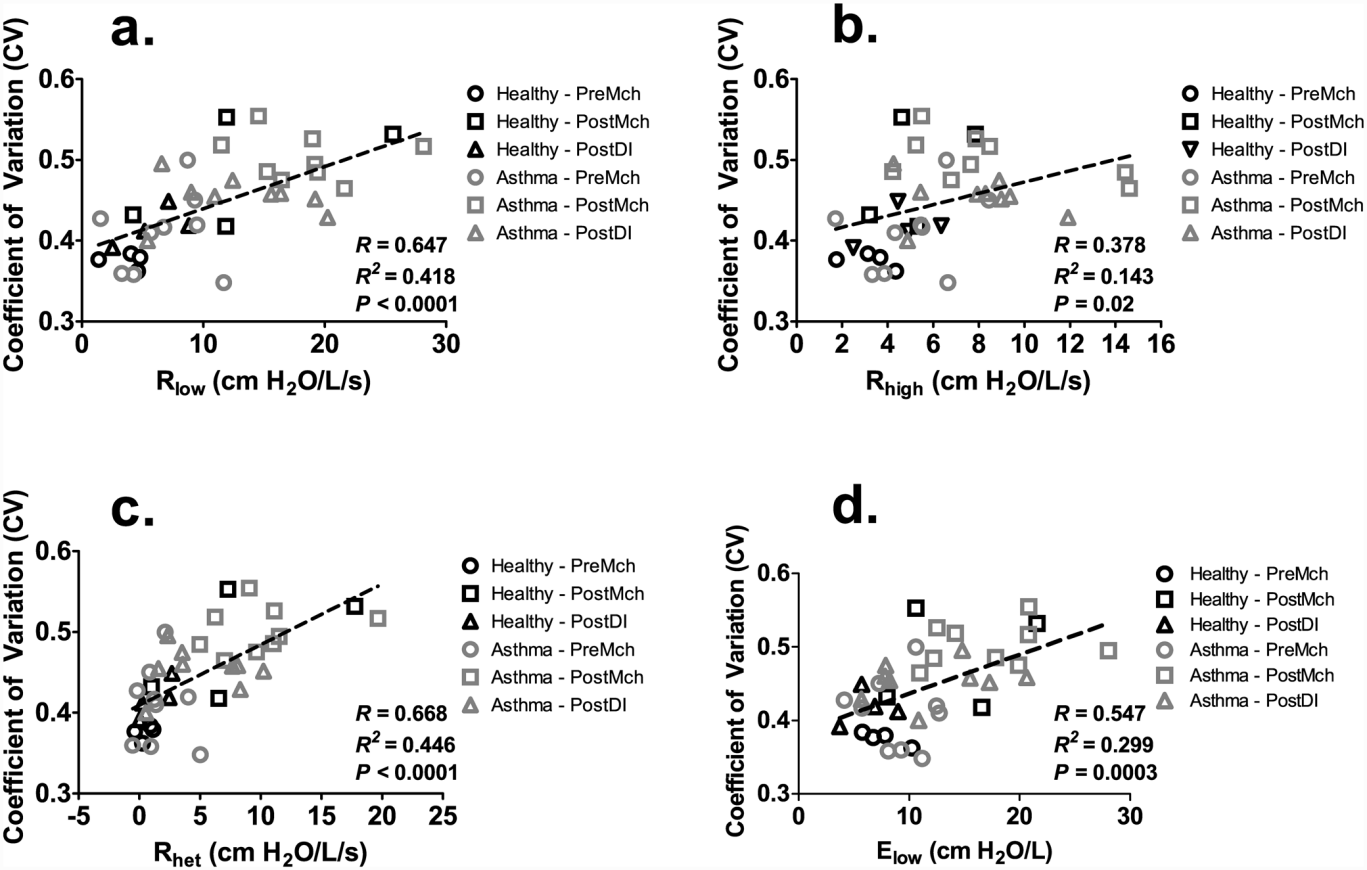
Association between CV and dynamic lung mechanics indices, R_low_ (a.), R_high_ (b.), R_het_ (c.), and E_low_ (d.). Note that while there was a significant correlation between CV and R_low_ (a.), R_het_ (c.), and E_low_ (d.), there was little correlation seen with R_high_ (b.) over all healthy subjects and mild-to-moderate asthmatics.

Correlation coefficients were calculated through a least squares linear regression between our image-derived CV of ventilation and measured dynamic lung mechanics, R_low_, R_high_, R_het_, and E_low_ over all subjects. The results are summarized in Fig 5. Over all the healthy and asthmatic subjects, CV was significantly correlated with R_low_ (*R* = 0.647, *P* < 0.0001), R_het_ (*R* = 0.668, *P* < 0.0001), and E_low_ (*R* = 0.547, *P* = 0.003). However, CV was found to have a low correlation to R_high_ (*R* = 0.378, *P* = 0.02). For the healthy subjects only, CV was significantly correlated to R_low_ (*R* = 0.775, *P* = 0.003), R_high_ (*R* = 0.581, *P* = 0.05), and R_het_ (*R* = 0.794, *P* = 0.002), but not significantly correlated to E_low_ (*R* = 0.543, *P* = 0.07). For the asthmatic subjects only, CV was significantly correlated to R_low_ (*R* = 0.541, *P* = 0.004), R_het_ (*R* = 0.573, *P* = 0.002), and E_low_ (*R* = 0.505, *P* = 0.007), but not significantly correlated to R_high_ (*R* = 0.257, *P* = 0.20).

### Relationship to AHR

**Fig 6:**
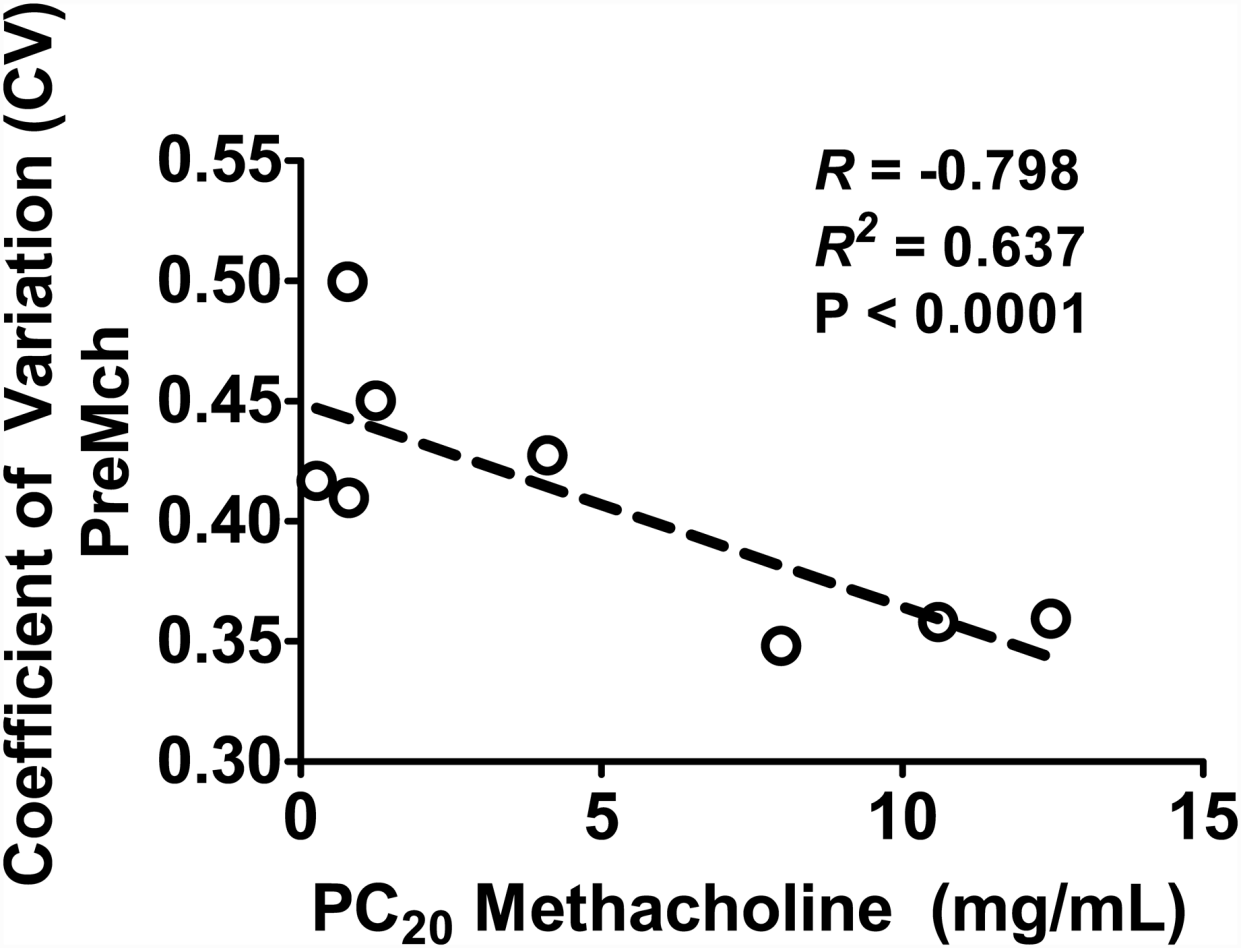
Baseline CV evaluated from HP ^3^He-MRI images is a predictor of AHR. There is a negative correlation between the baseline levels of CV and the PC_20_ dose in the asthmatic subjects. Subjects with a greater degree of baseline ventilation heterogeneity required a lower dose of agonist to achieve a 20% drop in FEV_1_ indicating the possibility of AHR when the subject is exposed to agonist.

Over the nine asthmatic subjects, AHR was evaluated from measurements of PC_20_. One asthmatic subject, however, exhibited a PC_20_ > 25 mg/ml and hence was excluded in our analysis. The results are illustrated in Fig 6. In the eight asthmatic subjects examined, PreMch CV showed a strong correlation to PC_20_ (*R* = -0.798, *P* < 0.0001). This correlation was not exhibited in PreMch measurements of dynamic lung mechanics, R_low_ (*R* = 0.16, *P* = 0.68), R_high_ (*R* = 0.29, *P* = 0.46), R_het_ (*R* = 0.038, *P* = 0.92), and E_low_ (*R* = 0.20, *P* = 0.60).

## Discussion

Ventilation heterogeneity continues to be a fundamental characteristic of asthma that has been shown to be a predictor of AHR [12, 13]. It has been extended to clinical applications of tracking responses to asthma therapy, specifically to the dose titration of inhaled corticosteroids [39]. Whereas indirect methods of determining ventilation heterogeneity through a MBNW technique is restricted by the lack of spatial information and its inability of detecting poorly ventilated areas [12–17], direct modalities of imaging run the risk of radiation exposure [18–22]. As a noninvasive imaging modality, HP ^3^He-MRI has emerged as a feasible alternative in visualizing the degree of ventilation heterogeneity.

### Relationship to Dynamic Lung Mechanics and AHR

Thus far, using qualitative methods of analysis, it has been shown that the number and size of ventilation defects rendered through HP ^3^He-MRI are directly correlated to the degree of clinical severity [25–27]. A majority of these defects remained persistent with time and with repeated bronchoconstriction [25, 26]. Since then, there have been a number of advancements in HP ^3^He-MRI with the emergence of quantitative methods to extract detailed structure-to-function relationships in asthma in both humans [23, 24, 30, 32] and animals [40–43]. For example, recent efforts have calculated a characteristic index to quantify the degree of ventilation heterogeneity [23, 30, 32]. What has not been established is if and how this index relates to the overall level of mechanical dysfunction and degree of AHR.

Our methods applied a semiautomated approach to the segmentation of ventilated airspaces [30] leading to a novel index to quantify the degree of heterogeneity throughout the imaged lung. Hence, we can quantitatively relate such heterogeneity to alterations in dynamic mechanical lung function sensitive to airways, tissues, and the degree of heterogeneous constriction [3, 20, 21, 33,34,36] as well as the overall reactivity (PC_20_) [4, 12, 15]. Similar to prior studies [7], we found that both healthy subjects that withhold DIs and mild-to-moderate asthmatics bronchoconstricted when exposed to airway agonist. In both healthy subjects and mild-to-moderate asthmatics, the degree of ventilation heterogeneity was correlated to the degree of mechanical heterogeneity of bronchoconstriction reflected by the indices of R_low_, R_het_, and E_low_ (Fig 5) but much less so to the parameter that reflects only the overall level of constriction averaged throughout the airway tree, R_high_. Both R_low_ and E_low_ reflect the mechanical properties of the lung periphery, particularly from contributions of the viscoelastic tissue properties and of the consequences of heterogeneous peripheral constriction [33, 34]. This finding was not evident from prior studies with MBNW [13], but that study only assessed mechanics at frequencies above 5 Hz which is not sensitive to the pattern of airway constriction whereas lower frequencies are. This latter frequency range permits more explicit insight on the separation of airway and tissue properties [33, 34]. For example, R_het_ which captures the degree of frequency dependence in dynamic lung resistance (R_L_) is consistent with heterogeneous constriction patterns [3, 36]. Together, these findings suggest a peripheral rather than central airway involvement in ventilation heterogeneity.

Most interesting was that utilizing a PC_20_ as an index for AHR, we demonstrated a strong link between baseline ventilation heterogeneity and the existence of AHR which was not seen in baseline measurements of dynamic lung mechanics. This is a new and potentially important finding. It suggests if the crucial component leading to enhanced AHR is the underlying airway conditions that have already created some level of abnormal ventilation patterns prior to provocation. Thus, if an otherwise asymptomatic asthmatic were to be exposed to a contractile stimulus (e.g., due to exposure to an allergen or enhanced inflammatory mediators), an existing slightly abnormal pattern of ventilation will enhance the subsequent degradation in mechanical lung function far more so than if this asthmatic exhibited a more uniform ventilation. Such a finding seems to suggest an exacerbation of the instability mechanisms proposed by Venegas *et al*. in symmetric airway trees [22] and by Leary *et al*. in asymmetric airway trees [44].

### Limitations

There are some limitations in our comparative analysis. To ensure safety of subjects who participated in our study, we set the maximum inhaled dose of Mch to 25 mg/ml. In some healthy subjects even this maximum dose did not elicit a 20% drop in FEV_1_. Thus, it is important to note that in our statistical comparisons, the degree of bronchoconstriction achieved was not the same among groups. Furthermore, we only recorded FEV_1_ at baseline and do not have measurements of FEV_1_ post-challenge with Mch. However, we did record measurements of R_low_, R_high_, R_het_ and E_low_ as well as calculated the CV in all subjects PostMch. There was not a statistically significant difference in PostMch between healthy and asthmatic subjects. Of the mild-to-moderate asthmatics, one asthmatic subject demonstrated PC_20_ which exceeded the maximum allowable dose of 25 mg/ml, likely reflecting a very mild form of asthma. For our statistical analysis, a repeated measure two-way ANOVA would have been the ideal test to compare our healthy and asthmatic populations. However, as our sample size was small, the power of this test turned out to be extremely low. Therefore, we opted to use both paired and unpaired comparisons with student’s *t*-tests in this study. All the results we report as being statistically significant, the power of the test is 0.8 or higher. Finally, our analytic measures of ventilation heterogeneity only quantified ventilated lung regions, namely regions that are not completely closed off. To include lung regions that are completely closed in severe bronchoconstriction, we recommend the use of ^1^H proton images for the segmentation of the entire thoracic cavity. However, such a method may pose some difficulty in extracting and distilling out the contribution of the trachea and major airways. In addition, due to technical limitations, imaging and lung mechanics measurements were not acquired in identical positions. Lung mechanics measurements vary with position where lung compliance has been found to decrease from sitting to supine while lung resistance has been found to increase [45]. However, when utilizing a forced oscillation technique to measure respiratory system resistance and elastance, these parameters do not change significantly in the sitting and supine position in past studies [18]. Ideally, for a direct one-to-one comparison, lung mechanics and imaging should be acquired in identical positions.

### Conclusion

In summary, in this study we advance a quantification of ventilation heterogeneity from HP ^3^He-MRI showing an agreement to measurements of dynamic lung mechanics. Our findings from dynamic lung mechanics were consistent with the notion of peripheral rather than central involvement of ventilation heterogeneity. Furthermore, our results demonstrate a strong link between the degree of baseline ventilation heterogeneity and AHR which may be of potential clinical use in evaluating for baseline asthma in instances where a Mch- challenge may not be suitable. In addition, recent clinical studies have shown that measurement of ventilation heterogeneity can be used as a predictor of the symptomatic response of asthmatic patients to changes in inhaled corticosteroid dose [39]. All-in-all, our technique is capable of delivering both structural and functional information that can be applied to tracking response to asthma therapy.

## Supporting Information

S1 Table CV and lung mechanics in healthy and asthmatic subjects. (DOCX)

## References

[1] Brusasco V, Crimi E, Barisione G, Spanevello A, Rodarte JR, Pellegrino R. Airway responsiveness to methacholine: effects of deep inhalations and airway inflammation. *J Appl Physiol*. 1999; 87(2):567–73. 10444614 10.1152/jappl.1999.87.2.567

[2] Kapsali T, Permutt S, Laube B, Scichilone N, Togias A. Potent bronchoprotective effect of deep inspiration and its absence in asthma. *J Appl Physiol*. 2000; 89(2):711–20. 10926658 10.1152/jappl.2000.89.2.711

[3] Lutchen KR, Jensen A, Atileh H, Kaczka DW, Israel E, Suki B, et al Airway constriction pattern is a central component of asthma severity: the role of deep inspirations. *Am J Respir Crit Care Med*. 2001; 164(2):207–15, 11463589 10.1164/ajrccm.164.2.2008119

[4] Mendonça NT, Kenyon J, LaPrad AS, Syeda SN, O’Connor GT, Lutchen KR. Airway resistance at maximum inhalation as a marker of asthma and airway hyperresponsiveness. *Respir Res*. 2011; 12:96 10.1186/1465-9921-12-96 21762517 PMC3143926

[5] Nadal JA, Tierney DF. Effect of a previous deep inspiration on airway resistance in man. *J Appl Physiol*. 1961; 16:717–9. 13727342 10.1152/jappl.1961.16.4.717

[6] Scichilone N, Permutt S, Togias A. The lack of the bronchoprotective and not the bronchodilatory ability of deep inspiration is associated with airway hyperresponsiveness. *Am J Respir Crit Care Med*. 2001; 163(2):413–9. 11179115 10.1164/ajrccm.163.2.2003119

[7] Black LD, Henderson AC, Atileh H, Israel E, Ingenito EP, Lutchen KR. Relating maximum airway dilation and subsequent reconstruction to reactivity in human lungs. *J Appl Physiol*. 2004; 96(5):1808–14. 14766781 10.1152/japplphysiol.01170.2003

[8] Skloot G, Permutt S, Togias A. Airway hyperresponsiveness in asthma: a problem of limited smooth muscle relaxation with inspiration. *J Clin Invest*. 1995; 96(5):2393–403. 7593627 10.1172/JCI118296 PMC185891

[9] King GG, Moore BJ, Seow CY, Paré PD. Airway narrowing associated with inhibition of deep inspiration during methacholine inhalation in asthmatics. *Am J Respir Crit Care Med*. 2001; 164(2):216–8. 11463590 10.1164/ajrccm.164.2.2006101

[10] King GG, Moore BJ, Seow CY, Paré PD. Time course of increased airway narrowing caused by inhibition of deep inspiration during methacholine challenge. *Am J Respir Crit Care Med*. 1999; 160(2):454–7. 10430713 10.1164/ajrccm.160.2.9804012

[11] Scichilone N, Marchese R, Soresi S, Interrante A, Togias A, Bellia V. Deep inspiration-induced changes in lung volume decrease with severity of asthma. *Respir Med*. 2007; 101(5):951–6. 17049828 10.1016/j.rmed.2006.09.009

[12] Downie SR, Salome CM, Verbanck S, Thompson B, Berend N, King GG. Ventilation heterogeneity is a major determinant of airway hyperresponsiveness in asthma, independent of airway inflammation. *Thorax*. 2007; 62(8):684–9. 17311839 10.1136/thx.2006.069682 PMC2117268

[13] Downie SR, Salome CM, Verbanck SA, Thompson BR, Berend N, King GG. Effect of methacholine on peripheral lung mechanics and ventilation heterogeneity in asthma. *J Appl Physiol* 2013; 114(6):770–7. 10.1152/japplphysiol.01198.2012 23372144

[14] Hardaker KM, Downie SR, Kermode JA, Farah CS, Brown NJ, Berend N, et al Predictors of airway hyperresponsiveness differ between old and young patients with asthma. Chest. 2011; 139(6):1395–401. 10.1378/chest.10-1839 21454398

[15] Farrow CE, Salome CM, Harris BE, Bailey DL, Bailey E, Berend N, et al Airway closure on imaging relates to airway hyperresponsiveness and peripheral airway disease in asthma. *J Appl Physiol*. 2012; 113(6):958–66. 10.1152/japplphysiol.01618.2011 22837168 PMC3472477

[16] Verbanck S, Schuermans D, Meysman M, Paiva M, Vincken W. Noninvasive assessment of airway alterations in smokers: the small airways revisited. *Am J Respir Crit Care Med*. 2004; 170(4):414–9. 15130906 10.1164/rccm.200401-037OC

[17] Mitchell JH, Hoffman EA, Tawhai MH. Relating indices of inert gas washout to localised bronchoconstriction. *Respir Physiol Neurobiol*. 2012; 183(3): 224–233. 10.1016/j.resp.2012.06.031 22771781 PMC3505678

[18] Kaminsky DA, Irvin CG, Lundblad LK, Thompson-Figueroa J, Klein J, Sullivan MJ, et al Heterogeneity of bronchoconstriction does not distinguish mild asthmatic subjects from healthy controls when supine. *J Appl Physiol*. 2008; 104(1):10–9. 17947503 10.1152/japplphysiol.00519.2007

[19] Harris RS, Fujii-Rios H, Winkler T, Musch G, Vidal Melo MF, Venegas JG. Ventilation defect formation in healthy and asthma subjects is determined by lung inflation. *PLoS One*. 2012; 7(12):e53216 10.1371/journal.pone.0053216 23285270 PMC3532117

[20] Tgavalekos NT, Tawhai M, Harris RS, Musch G, Vidal-Melo M, Venegas JG, et al Identifying airways responsible for heterogeneous ventilation and mechanical dysfunction in asthma: an image functional modeling approach. *J Appl Physiol*. 2005; 99(6):2388–97. 16081622 10.1152/japplphysiol.00391.2005

[21] Tgavalekos NT, Musch G, Harris RS, Vidal Melo MF, Winkler T, Schroeder T, et al Relationship between airway narrowing, patchy ventilation and lung mechanics in asthmatics. *Eur Respir J*. 2007; 29(6):1174–81. 17360726 10.1183/09031936.00113606

[22] Venegas JG, Winkler T, Musch G, Vidal Melo MF, Layfield D, Tgavalekos N, et al Self-organized patchiness in asthma as a prelude to catastrophic shifts. *Nature*. 2005; 434(7034):777–82. 15772676 10.1038/nature03490

[23] Campana L, Kenyon J, Zhalehdoust-Sani S, Tzeng YS, Sun Y, Albert MS, et al Probing airway conditions governing ventilation defects in asthma via hyperpolarized MRI image functional modeling. *J Appl Physiol*. 2009; 106(4):1293–300. 10.1152/japplphysiol.91428.2008 19213937 PMC2698646

[24] Costella S, Kirby M, Maksym GN, McCormack DG, Paterson NA, Parraga G. Regional pulmonary response to a methacholine challenge using hyperpolarized 3He magnetic resonance imaging. Respirology. 2012; 17(8):1237–46. 10.1111/j.1440-1843.2012.02250.x 22889229

[25] de Lange EE, Altes TA, Patrie JT, Battiston JJ, Juersivich AP, Mugler JP 3rd, et al Change in regional airflow obstruction over time in the lungs of patients with asthma: evaluation with 3He MR imaging. Radiology. 2009; 250(2):567–75. 10.1148/radiol.2502080188 19188325

[26] de Lange EE, Altes TA, Patrie JT, Parmar J, Brookeman JR, Mugler JP 3rd, et al The variability of regional airflow obstruction within the lungs of patients with asthma: assessment with hyperpolarized helium-3 magnetic resonance imaging. *J Allergy Clin Immunol*. 2007; 119(5):1072–8. 17353032 10.1016/j.jaci.2006.12.659

[27] de Lange EE, Altes TA, Patrie JT, Gaare JD, Knake JJ, Mugler JP 3rd, et al Evaluation of asthma with hyperpolarized helium-3 MRI: correlation with clinical severity and spirometry. Chest. 2006; 130(4):1055–62. 17035438 10.1378/chest.130.4.1055

[28] Kauczor HU, Markstaller K, Puderbach M, Lill J, Eberle B, Hanisch G, et al Volumetry of ventilated airspaces by 3He MRI: preliminary results. Invest Radiol. 2001; 36(2):110–4. 11224759 10.1097/00004424-200102000-00007

[29] Lewis TA, Tzeng YS, McKinstry EL, Tooker AC, Hong K, Sun Y, et al Quantification of airway diameters and 3D airway tree rendering from dynamic hyperpolarized ^3^He magnetic resonance imaging. *Magn Reson Med*. 2005; 53(2):474–8. 15678546 10.1002/mrm.20349 PMC2930613

[30] Lui JK, LaPrad AS, Parameswaran H, Sun Y, Albert MS, Lutchen KR. Semiautomatic segmentation of ventilated airspaces in healthy and asthmatics using hyperpolarized 3He MRI. *Comput Math Methods Med*. 2013; 2013:624683 10.1155/2013/624683 23606904 PMC3626384

[31] Samee S, Altes T, Powers P, de Lange EE, Knight-Scott J, Rakes G, et al Imaging the lungs in asthmatic patients by using hyperpolarized helium-3 magnetic resonance: assessment of response to methacholine and exercise challenge. *J Allergy Clin Immunol*. 2003; 111(6):1205–11. 12789218 10.1067/mai.2003.1544

[32] Tzeng YS, Lutchen KR, Albert MS. The difference in ventilation heterogeneity between asthmatic and healthy subjects quantified using hyperpolarized ^3^He MRI. *J Appl Physiol*. 2009; 106(3):813–22. 10.1152/japplphysiol.01133.2007 19023025

[33] Kaczka DW, Ingenito EP, Suki B, Lutchen KR. Partitioning airway and lung tissue resistances in humans: effects of bronchoconstriction. *J Appl Physiol*. 1997; 82(5):1531–41. 9134903 10.1152/jappl.1997.82.5.1531

[34] Lutchen KR, Greenstein JL, Suki B. How inhomogeneities and airway walls affect frequency dependence and separation of airway and tissue properties. *J Appl Physiol*. 1996; 80(5):1696–707. 8727557 10.1152/jappl.1996.80.5.1696

[35] Lutchen KR, Yang K, Kaczka DW, Suki B. Optimal ventilation waveforms for estimating low-frequency respiratory impedance. *J Appl Physiol*. 1993; 75(1):478–88. 8376299 10.1152/jappl.1993.75.1.478

[36] Lutchen KR, Gillis H. Relationship between heterogeneous changes in airway morphometry and lung resistance and elastance. *J Appl Physiol*. 1997; 83(4):1192–201. 9338428 10.1152/jappl.1997.83.4.1192

[37] Gudbjartsson H, Patz S. The rician distribution of noisy MRI data. *Magn Reson Med*. 1995; 34:910–14. 8598820 10.1002/mrm.1910340618 PMC2254141

[38] Henkelman RM. Measurement of signal intensities in the presence of noise in MR images. Med Phys. 1985; 12(2):232–33. 4000083 10.1118/1.595711

[39] Farah CS, King GG, Brown NJ, Peters MJ, Berend N, Salome CM. Ventilation heterogeneity predicts asthma control in adults following inhaled corticosteroid dose titration. *J Allergy Clin Immunol*. 2012; 130(1):61–8. 10.1016/j.jaci.2012.02.015 22460065

[40] Driehuys B, Walker J, Pollaro J, Cofer GP, Mistry N, Schwartz D, et al ^3^He MRI in mouse models of asthma. *Magn Reson Med*. 2007; 58(5):893–900. 17969115 10.1002/mrm.21306 PMC2746053

[41] Lundblad LK, Thompson-Figueroa J, Allen GB, Rinaldi L, Norton RJ, Irvin CG, et al Airway hyperresponsiveness in allergically inflamed mice: the role of airway closure. *Am J Respir Crit Care Med*. 2007; 175(8):768–74. 17255559 10.1164/rccm.200610-1410OC PMC1899295

[42] Mistry NN, Thomas A, Kaushik SS, Johnson GA, Driehuys B. Quantitative analysis of hyperpolarized ^3^He ventilation changes in mice challenged with methacholine. *Magn Reson Med*. 2010; 63(3):658–66. 10.1002/mrm.22311 20187176 PMC2872792

[43] Thomas AC, Kaushik SS, Nouls J, Potts EN, Slipetz DM, Foster WM, et al Effects of corticosteroid treatment on airway inflammation, mechanics, and hyperpolarized 3He magnetic resonance imaging in an allergic mouse model. *J Appl Physiol*. 2012; 112(9):1437–44. 10.1152/japplphysiol.01293.2011 22241062 PMC3362235

[44] Leary D, Winkler T, Braune A, Maksym GN. Effects of airway tree asymmetry on the emergence and spatial persistence of ventilation defects. *J Appl Physiol*. 2014; 117(4):353–62. 10.1152/japplphysiol.00881.2013 24947031 PMC4137237

[45] Behrakis PK, Baydur A, Jaeger MJ, Milic-Emili J. Lung mechanics in sitting and horizontal body positions. *Chest*. 1983; 83(4):643–6. 6831953 10.1378/chest.83.4.643

