# Supplementary material for: Linking Ventilation Heterogeneity Quantified via Hyperpolarized 3He MRI to Dynamic Lung Mechanics and Airway Hyperresponsiveness
Source: PLoS One. 2015 Nov 16;10(11):e0142738. doi: 10.1371/journal.pone.0142738 (PMC4646346; doi:10.1371/journal.pone.0142738)
Supplement: S1 Table — (DOCX) [file pone.0142738.s001.docx]

**Supplemental Table 1. CV and lung mechanics in healthy and asthmatic subjects.**

|  | Condition | CV | Rlow | Rhigh | Rhet | Elow |
| --- | --- | --- | --- | --- | --- | --- |
| H1 |  |  |  |  |  |  |
|  | PreMch | 0.3839 | 4.0595 | 3.1388 | 0.9207 | 5.7722 |
|  | PostMch | 0.5527 | 11.9366 | 4.6227 | 7.3139 | 10.6013 |
|  | PostDI | 0.4488 | 7.1345 | 4.4511 | 2.6834 | 5.6859 |
| H2 |  |  |  |  |  |  |
|  | PreMch | 0.3792 | 4.7995 | 3.6714 | 1.1281 | 7.8234 |
|  | PostMch | 0.5316 | 25.6057 | 7.8587 | 17.7469 | 21.5967 |
|  | PostDI | 0.4189 | 8.8124 | 6.3446 | 2.4678 | 6.8923 |
| H3 |  |  |  |  |  |  |
|  | PreMch | 0.3766 | 1.3963 | 1.7573 | -0.3610 | 6.7567 |
|  | PostMch | 0.432 | 4.2264 | 3.2135 | 1.0130 | 7.9905 |
|  | PostDI | 0.3915 | 2.5282 | 2.4884 | 0.0398 | 3.7001 |
| H4 |  |  |  |  |  |  |
|  | PreMch | 0.3623 | 4.6205 | 4.3532 | 0.2673 | 10.2652 |
|  | PostMch | 0.4177 | 11.8668 | 5.3229 | 6.5439 | 16.5866 |
|  | PostDI | 0.4118 | 5.1690 | 4.9171 | 0.2519 | 8.9883 |
| A1 |  |  |  |  |  |  |
|  | PreMch | 0.3581 | 4.2819 | 3.3230 | 0.9589 | 8.1044 |
|  | PostMch | 0.5543 | 14.5593 | 5.4923 | 9.0670 | 20.8391 |
|  | PostDI | 0.4953 | 6.5892 | 4.2525 | 2.3368 | 14.8058 |
| A2 |  |  |  |  |  |  |
|  | PreMch | 0.4194 | 9.4973 | 5.4566 | 4.0407 | 12.4977 |
|  | PostMch | 0.475 | 16.4473 | 6.7860 | 9.6613 | 19.9226 |
|  | PostDI | 0.4576 | 15.5979 | 7.9284 | 7.6696 | 15.5051 |
| A3 |  |  |  |  |  |  |
|  | PreMch | 0.4274 | 1.5523 | 1.7124 | -0.1601 | 4.1217 |
|  | PostMch | 0.5183 | 11.4925 | 5.2360 | 6.2565 | 14.1983 |
|  | PostDI | 0.4601 | 9.0143 | 5.4437 | 3.5706 | 7.8082 |
| A4 |  |  |  |  |  |  |
|  | PreMch | 0.4098 | 5.6953 | 4.3299 | 1.3654 | 12.7215 |
|  | PostMch | 0.4945 | 19.1747 | 7.6459 | 11.5288 | 28.0518 |
|  | PostDI | 0.4583 | 16.3745 | 8.2917 | 8.0828 | 20.6529 |
| A5 |  |  |  |  |  |  |
|  | PreMch | 0.3481 | 11.6820 | 6.6468 | 5.0351 | 11.1713 |
|  | PostMch | 0.5261 | 18.9967 | 7.8769 | 11.1198 | 12.5149 |
|  | PostDI | 0.4748 | 12.4165 | 8.8923 | 3.5242 | 7.8745 |
| A6 |  |  |  |  |  |  |
|  | PreMch | 0.4997 | 8.7158 | 6.5823 | 2.1335 | 10.6133 |
|  | PostMch | 0.5167 | 28.1416 | 8.4837 | 19.6578 | 20.8123 |
|  | PostDI | 0.4515 | 19.2147 | 8.9879 | 10.2268 | 17.2730 |
| A7 |  |  |  |  |  |  |
|  | PreMch | 0.4167 | 6.7570 | 5.5132 | 1.2438 | 5.7409 |
|  | PostMch | 0.4645 | 21.6290 | 14.6100 | 7.0190 | 10.9668 |
|  | PostDI | 0.4287 | 20.2291 | 11.9185 | 8.3107 | 5.6773 |
| A8 |  |  |  |  |  |  |
|  | PreMch | 0.4502 | 9.3005 | 8.4450 | 0.8554 | 7.2925 |
|  | PostMch | 0.4845 | 19.4118 | 14.4326 | 4.9792 | 12.2284 |
|  | PostDI | 0.4547 | 10.9471 | 9.3652 | 1.5819 | 8.2238 |
| A9 |  |  |  |  |  |  |
|  | PreMch | 0.3595 | 3.3053 | 3.8532 | -0.5479 | 9.2764 |
|  | PostMch | 0.4855 | 15.2653 | 4.2278 | 11.0375 | 17.8396 |
|  | PostDI | 0.4 | 5.4107 | 4.8751 | 0.5355 | 10.8647 |
